# Chemical Inhibitors of Non-Homologous End Joining Increase Targeted Construct Integration in *Cryptococcus neoformans*

**DOI:** 10.1371/journal.pone.0163049

**Published:** 2016-09-19

**Authors:** Samantha D. M. Arras, James A. Fraser

**Affiliations:** Australian Infectious Diseases Research Centre, School of Chemistry & Molecular Biosciences, The University of Queensland, St Lucia, QLD 4072 Australia; Tulane University Health Sciences Center, UNITED STATES

## Abstract

The development of a biolistic transformation protocol for *Cryptococcus neoformans* over 25 years ago ushered in a new era of molecular characterization of virulence in this previously intractable fungal pathogen. However, due to the low rate of homologous recombination in this species, the process of creating targeted gene deletions using biolistic transformation remains inefficient. To overcome the corresponding difficulty achieving molecular genetic modifications, members of the *Cryptococcus* community have investigated the use of specific genetic backgrounds or construct design strategies aimed at reducing ectopic construct integration via non-homologous end joining (NHEJ). One such approach involves deletion of components of the NHEJ-associated Ku heterodimer. While this strategy increases homologous recombination to nearly 100%, it also restricts strain generation to a *ku80Δ* genetic background and requires subsequent complex mating procedures to reestablish wild-type DNA repair. In this study, we have investigated the ability of known inhibitors of mammalian NHEJ to transiently phenocopy the *C*. *neoformans* Ku deletion strains. Testing of eight candidate inhibitors revealed a range of efficacies in *C*. *neoformans*, with the most promising compound (W7) routinely increasing the rate of gene deletion to over 50%. We have successfully employed multiple inhibitors to reproducibly enhance the deletion rate at multiple loci, demonstrating a new, easily applied methodology to expedite acquisition of precise genetic alterations in *C*. *neoformans*. Based on this success, we anticipate that the use of these inhibitors will not only become widespread in the *Cryptococcus* community, but may also find use in other fungal species as well.

## Introduction

The discovery of the process of transformation was key to the development of the field of molecular genetics. The first evidence that genetic information could be introduced into a cell came in 1928 when Frederick Griffith discovered that a “transforming factor” could make a harmless strain of *Streptococcus pneumoniae* virulent after being exposed to a heat-killed virulent strain, giving rise to the term transformation [1]. It was not until 1944 that Avery and colleagues used transformation to prove that this factor was DNA [2]. The era of eukaryotic molecular genetics began over thirty years later when Hinnen and colleagues employed transformation in brewer’s yeast to integrate a plasmid into the *Saccharomyces cerevisiae* genome [3]. Beggs subsequently demonstrated that *S*. *cerevisiae* could maintain a plasmid carrying the 2μ origin of replication without the need for integration [4]. These discoveries established *S*. *cerevisiae* as the premier eukaryotic model for molecular genetics. Transformation protocols were subsequently developed for *Neurospora crassa* [5] and *Aspergillus nidulans* [6], and over the following decades, the development of transformation protocols made many previously intractable species easier to study.

*Cryptococcus neoformans* is one such species. Found worldwide in association with bird guano, *C*. *neoformans* primarily causes disease in immunocompromised individuals, disseminating *via* the lungs to cause life-threatening meningoencephalitis; it is classified as an AIDS-defining illness. In developed countries, the mortality rate is as high as 20% [7], but in developing countries where there is limited availability of treatment, infection can result in close to 100% mortality [8, 9]. While transformation of *C*. *neoformans via* electroporation was achieved over 25 years ago [10], the technique was not widely adopted due to its extremely low homologous integration efficiency and the instability of transformants. It was not until the development of a biolistic protocol in 1993 that molecular genetic manipulation in this organism became routine [11]. Although biolistic technology is now widely employed, creating gene deletions in *C*. *neoformans* can still be difficult due to the poor reproducibility of the biolistic technique and low levels of integration *via* homologous recombination [11–13]; the majority of transformants are either ectopic integrants or unstable [14].

Upon introduction of genetic material into a cell *via* transformation there are, broadly, four possible fates. First, the exogenous DNA may be maintained extrachromosomally in the form of a plasmid or minichromosome, provided this is possible in the host species and the DNA sequence is appropriate. Second, the foreign DNA may simply be degraded by the host machinery. Third, the exogenous DNA may integrate into the genome in a targeted manner *via* homologous recombination, and lastly, the exogenous DNA may integrate at a random site in the genome. These two mechanisms of integration into the genome occur by very different mechanisms. Homologous recombination occurs through crossing over where DNA sequences are exchanged between two similar molecules of DNA; this method is the basis for creating targeted gene deletions. While creating gene deletions *via* homologous recombination occurs readily in species such as *S*. *cerevisiae*, it occurs at a lower frequency in many organisms, including *C*. *neoformans*. These species instead tend to predominantly employ non-homologous end joining (NHEJ) and integrate the transformed DNA into a random location in the genome.

In eukaryotes, NHEJ begins with the DNA-dependent protein kinase heterodimeric regulatory factor Ku70-Ku80 forming a link between two broken DNA ends and acting to structurally support, align and protect them from further degradation [15]. In humans, the Ku70-Ku80 heterodimer recruits the catalytic subunit DNA-PK_cs_, which is thought to induce conformational changes that allow end-processing enzymes to access the DNA ends [16–18]; in *S*. *cerevisiae*, a complex consisting of Mre11, Rad50 and Xrs2 is thought to perform this function instead [19]. In both species the process of NHEJ is completed when the DNA ligase IV-Xrcc4 complex ligates the broken ends of the DNA back together [16, 18–20].

Homologous recombination and NHEJ compete directly against one another to incorporate foreign DNA into the genome. Unfortunately for molecular geneticists, in most species NHEJ usually triumphs over homologous recombination, frustrating researcher’s attempts to make precise genetic modifications. One solution to this problem is to enhance homologous recombination by creating mutants defective in NHEJ [21]. Deletion of the *ku* genes in *N*. *crassa* [22], *Kluyveromyces lactis* [23], and *A*. *nidulans* [24] have all resulted in increased gene deletion success, with targeted integration rates exceeding 90%.

Ku deletion mutants have also been generated in *C*. *neoformans*, with mutants exhibiting almost 100% homologous recombination following biolistic transformation [13]. Lin *et al*. have also shown that the use of a *ku80Δ* mutant strain increases the rate of homologous integration when using electroporation up to 75%, making this previously superseded technique a viable alternative to biolistic transformation provided the recipient strain is a *ku* mutant [25]. Unfortunately, using Ku deletion mutants to ensure targeted integration subsequently requires sexual crosses (both time consuming and technically difficult) with a wild-type partner to restore NHEJ because loss of the Ku heterodimer alters virulence. Expression of *ku80* is increased during infection in a human host [26], and a *ku80Δ* mutant is less successful in a competition model of murine infection [27]. Consequently, while useful, Ku deletion strains have not been adopted for widespread use by the *C*. *neoformans* community.

Here we describe an alternate strategy to enhance homologous integration in *C*. *neoformans* by transient inhibition of NHEJ with the aid of chemical inhibitors. Using a range of candidate drugs that have been shown to inhibit NHEJ in mammalian cell lines, we have successfully identified compounds that enable rates of homologous integration consistently greater than 50%. The efficacy of these compounds in *C*. *neoformans* provides an attractive tool for the community as they provide the ability to increase homologous integration in a strain-independent fashion, making molecular genetic manipulations easier to achieve.

## Methods

### Strains and growth conditions

*C*. *neoformans* var. *grubii* type strain H99 was stored in 15% glycerol at -80°C until use, at which point it was maintained on YPD at 4°C for a maximum of two weeks; this strain was used for all transformations. *C*. *neoformans* was cultured in liquid (2% bacto-peptone, 1% yeast extract, and 2% glucose (Sigma, USA)) or solid (2% agar added) YPD media at 30°C. Biolistic transformations were performed on solid YPD media supplemented with 1 M sorbitol (Sigma, USA), and transformants were selected on solid YPD medium containing 100 μg/mL G418 (Sigma, USA). Adenine auxotrophy was detected on YNB media (0.45% yeast nitrogen base w/o amino acids and ammonium sulfate, 2% glucose, 0.5% ammonium sulfate, 2% agar (Sigma, USA)), and melanin production on L-DOPA media (Sigma, USA) [28].

### Checkerboard assays

The inhibitors NU7026, NU7441, AG14361 (Selleckchem, USA), mirin (Life Chemicals, Canada), SCR7 (DSK Biopharma Products, USA), W7 hydrochloride (TCI UK Fine Chemicals, UK), vanillin, chlorpromazine, as well as the DNA damaging agents phleomycin, hydroxyurea and 6-mercaptopurine riboside (Sigma, USA) were stored at -20°C until use. Stock solutions and serial two-fold dilutions of each drug were prepared immediately prior to MIC testing according to the recommendations of CLSI (CLSI M27-A2) modified for *C*. *neoformans* [29, 30]. For the Fractional Inhibitory Concentration (FIC) assays [31], inhibitors were serially diluted along the abscissa of a 96 well plate, while the DNA damaging agents were diluted along the ordinate. Plates were incubated at 35°C and visually scored at 24 and 48 hr. All MIC and FIC assays were performed in duplicate.

MIC was defined by the lowest concentration of a specific drug that inhibits all visual growth, and was used to determine a ΣFIC value for each combination set. The ΣFIC was calculated as follows: ΣFIC = FIC A + FIC B, where FIC A equals the MIC of the DNA damaging agent in the combination divided by the MIC of the DNA damaging agent alone, and FIC B equals the MIC of the inhibitor in the combination divided by the MIC of the inhibitor alone. The combination was considered strongly synergistic when the ΣFIC value is <0.5, weakly synergistic when ΣFIC is 0.6 to 1.0, additive when ΣFIC is 1.0 to 2.0 and antagonistic when the ΣFIC is >2 [31].

### Gene deletion construct generation

Primers used in this study are listed in S1 Table; all PCR was performed using Phusion DNA Polymerase (New England Biolabs, USA). A deletion construct for the *ADE2* gene was generated as per Arras *et al*. [14]. Briefly, the construct was prepared using overlap PCR, employing primers UQ1439 and UQ1442 to join the *ADE2* 5’ region (primers UQ1439 and UQ1440), the G418 resistance marker *NEO* (UQ234 and UQ235) and the *ADE2* 3’ region (UQ1441 and UQ1442). For *LAC1*, primers UQ3686 and UQ3691 were employed to join the *LAC1* 5’ region (primers UQ3686 and UQ3687), the G418 resistance marker (UQ3688 and UQ3689) and the *LAC1* 3’ region (UQ3690 and UQ3691)(S1 Table); H99 genomic DNA was used as the template for *ADE2* and *LAC1*, and the plasmid pJAF1 for *NEO* [32].

### Transformations

Transformations were performed using a procedure modified from Tofaletti *et al*. and Davidson *et al* [11, 12]. On the day of transformation, fresh YPD agar plates supplemented with 1 M sorbitol and the tested inhibitor were prepared and dried. A 50 mL *C*. *neoformans* culture grown at 30°C for 16 hr was pelleted by centrifugation, washed in dH_2_O, centrifuged and the pellet resuspended in 500 μL dH_2_O. Approximately 10^7^ cells were plated onto the inhibitor media, dried in a biosafety cabinet, then incubated for 4 hr at 30°C.

For transformation, 0.25 g of 0.6 μm Gold Microcarriers (Bio-Rad No. 165–2262) were resuspended in 2 mL 100% ethanol. Transforming DNA samples were prepared by mixing 10 μL Gold Microcarriers (Bio-Rad, USA), 10 μL of construct DNA (1 μg/μL), 10 μL 2.5 M CaCl_2_ (Sigma, USA) and 2 μl fresh 1 M spermidine (Sigma, USA), then incubated for 5 min at 20°C. Following centrifugation and washing in 500 μL 100% ethanol, the sample was pelleted and resuspended in 5 μL 100% ethanol.

For each transformation a Macrocarrier disk (No. 165–2335, Bio-Rad, USA) and a Stopping Screen (No. 165–2336, Bio-Rad, USA) were sterilized in 100% ethanol and dried in a Petri dish. Transforming DNA was pipetted onto the center of the disk, dried then inserted into the Macrocarrier Holder of a Bio-Rad Biolistic PDS-1000/He Particle Delivery System (No. 165–2257, Bio-Rad, USA). A 1,350 psi Rupture Disk (No. 165–2330, Bio-Rad, USA) sterilized in 70% isopropanol was inserted into the Rupture Disk Retaining Cap. The sterilized Stopping Screen was inserted into the Microcarrier Launch Assembly, the Macrocarrier Holder attached with the Macrocarrier Cover Lid, and placed in position one in the PDS-1000/He chamber. Transformation was performed with the *C*. *neoformans* plate in position three, bombarded at 1,350 psi under a vacuum of 27 in.Hg.

After recovery at 30°C for 4 hr, 2.5 mL dH_2_O was added to each plate, the cells suspended with a 1 mL pipette tip, and spread over five YPD plates containing 100 μg/mL G418. Transformants appeared after 3–5 days incubated at 30°C.

### Mutant verification

Correct *ade2Δ* integrants were initially identified *via* their pink coloration on YPD media and adenine auxotrophy on YNB media. Representative pink transformants were selected for Southern blot analyses to validate correct integration of the *ADE2* deletion cassette using the UQ1439 and UQ1442 PCR product as a probe. Correct *lac1Δ* integrants were identified by their lack of melanization on L-DOPA media, with random transformants verified by Southern blot using the UQ3686 and UQ3691 PCR product as a probe. All transformations were performed in triplicate and only transformations that resulted in 10 or more colonies were included in subsequent analyses. To determine significance of correct integration rates between conditions, two-tailed *t*-tests were completed in GraphPad Prism Version 6.0c (GraphPad Software, USA). *P*-values of <0.05 were considered statistically significant.

## Results

### Identification of potential inhibitors of *C*. *neoformans* NHEJ

**Fig 1:**
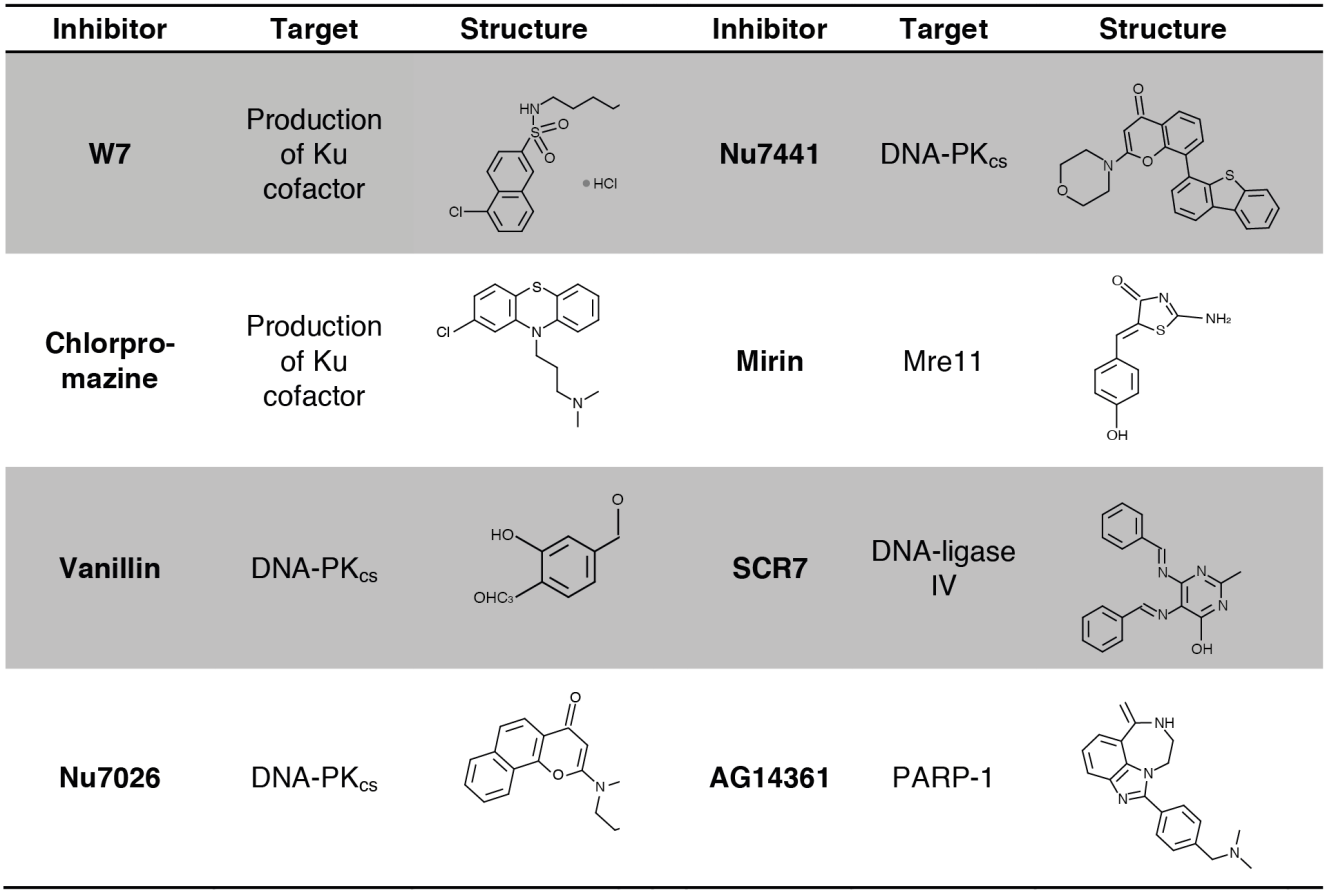
Inhibitors used in this study. Eight chemical inhibitors were employed in this study. Depicted above are their chemical structures and their targets in mammalian cell lines.

Given the success in increasing homologous integration in many fungal species by disrupting NHEJ *via* deletion of genes encoding the Ku proteins [13, 33], we hypothesized that it might be possible to achieve similar results by instead transiently inhibiting key proteins involved in NHEJ repair. Through literature searches we were able to identify seven candidate chemical compounds that are known to inhibit different aspects of mammalian NHEJ, are cost effective, and are readily available on the market (Fig 1).

Therapeutic inhibition of the mammalian DNA-dependent protein kinase heterodimeric regulatory factor (the Ku heterodimer) can be achieved through depletion of its cofactor inositol hexakisphosphate (InsP_6_) *via* the calmodulin antagonists N-(6-aminohexyl)-5-chloro-1-naphthalenesulfonamide (W7) and 2-chloro-10-(3-dimethylaminopropyl)phenothiazine hydrochloride (chlorpromazine) [34]. Likewise, inhibition of the catalytic subunit of the mammalian DNA-dependent protein kinase catalytic subunit (DNA-PK_cs_) is also possible; the *Vanilla planifolia*-derived phenolic aldehyde vanillin, the benzochromenone 2-(morpholin-4-yl)-benzo[h]chomen-4-one (Nu7026) and the aryl-substituted chromenone 8-dibenzothiophen-4-yl-2-morpholin-4-yl-chromen-4-one (Nu7441) all inhibit mammalian DNA-PK, and have been investigated as anticancer agents [35–37]. Z-5-(4-hydroxybenzylidene)-2-imino-1,3-thiazolidin-4-one (mirin) is a known inhibitor of Mre11, and 5,6-bis(((E)-benzylidene)amino)-2-thioxo-2,3-dihydropyrimidin-4(1H)-one (SCR7) targets DNA ligase IV [38–41].

In addition to these known NHEJ inhibitors, we also chose to study a compound not directly associated with NHEJ—the poly(ADP-ribose) polymerase-1 (PARP-1) inhibitor 1-(4-((dimethylamino)methyl)phenyl)-8,9-dihydro-2,7,9a-triazabenzo[cd]azulen-6(7H)-one (AG14361), a tricyclic benzimidazole that inhibits single strand DNA repair. As mammalian cells lacking PARP-1 have been shown to have an increased frequency of homologous recombination [42], we reasoned that treatment with an inhibitor of this enzyme may also lead to an increase in homologous recombination in *C*. *neoformans*.

To our knowledge, none of these eight drugs have ever been tested against *C*. *neoformans*.

### Growth inhibition of *C*. *neoformans* by inhibitors of mammalian NHEJ

It has previously been demonstrated that the eight chosen inhibitors abolish growth in mammalian cell lines at high concentrations, however it is unknown if they exhibit toxicity against *C*. *neoformans*.

To determine the minimum inhibitory concentration (MIC) for each compound in *C*. *neoformans*, we based our initial concentration ranges on MIC values identified in previous studies against mammalian cell lines (Table 1). Initial tests revealed that growth of *C*. *neoformans* var. *grubii* type strain H99, the strain in which the vast majority of molecular genetics is performed in this species, is unaffected at the concentrations toxic to mammalian cell culture. Following testing with increasing concentrations, growth inhibition was eventually observed at concentrations substantially higher than that tolerated by mammalian cells (Table 1).

**Table 1:** MIC values for mammalian cell lines and for *C*. *neoformans*. Mammalian MICs were originally published in nM and have been converted to μg/mL in this table to enable comparison to the *C*. *neoformans* data.

Inhibitor	Mammalian MIC (μg/mL)	Cell line	Reference	*C*. *neoformans* MIC (μg/mL)
**W7**	10.6	Human HeLa cells	[43]	75
**Chlorpromazine**	35.5	Human 293T cells	[34]	160
**Vanillin**	45.6	Human GM00558 lymphoma cells	[35]	750
**Nu7026**	2.8	Chinese hamster V3YAC cells	[42]	125
**Nu7441**	0.206	Human SW620 cells	[37]	250
**Mirin**	2.2	Chinese hamster ovary cells	[39]	320
**SCR7**	16.6	Mouse embryos	[44]	720
**AG14361**	0.005	Human SW620 cells	[45]	250

### Synergy between inhibitors and DNA damaging agents

While our MIC assays showed that the candidate NHEJ inhibitors were toxic to *C*. *neoformans* in sufficient concentrations, it did not prove that the observed effects were mediated *via* DNA repair-mediated processes. To provide this evidence we investigated their synergy with known DNA damaging agents; if the observed toxicity was due to inhibition of DNA repair, treatment with the inhibitors should increase sensitivity to DNA damaging agents.

The growth of strain H99 was tested in a checkerboard assay, with increasing concentrations of each DNA damaging agent tested in combination with increasing concentrations of each inhibitor to determine the factorial inhibition concentration (FIC). The FIC index is based on the Loewe additive zero-interaction theory, which states that a drug cannot interact with itself and therefore a drug combination will always be additive with an index of 1 [46]. If the index fluctuates lower indicating synergy it is because more drug would be required in order to produce the same affect of the drugs alone, and the reverse also. As the DNA damaging agents are designed to induce double strand DNA breaks and the NHEJ proteins should be involved in the repair of these breaks, we hypothesized that if a synergistic effect was observed the tested compounds were potentially inhibiting *C*. *neoformans* NHEJ.

As DNA damaging agents vary in their effectiveness and method of action, we selected three for our FIC assays. Phleomycin attracts hydrogen atoms from deoxyribose, producing apurinic and apyrimidinic sites that enable single strand breaks, which can in turn become double strand breaks [47]. Hydroxyurea depletes cells of dNTPs *via* inhibition of ribonucleotide reductase, resulting in stalled replication forks that can collapse into double strand breaks [48]. 6-mercaptopurine is converted into thioinosinic acid, which perturbs purine metabolism and results in double strand breaks [49]. As a starting point for FICs, MIC values were determined for the DNA damaging agents: 10 μg/mL for phleomycin, 1500 μg/mL for mercaptopurine, and 1500 μg/mL for hydroxyurea.

An FIC is considered synergistic when the ΣFIC is <0.5 and weakly synergistic when ΣFIC is >0.5 to <1.0. All of the inhibitors tested showed a weak synergistic effect against at least one of the DNA damaging agents tested. Nu7026, SCR7 and chlorpromazine exhibited synergy with all three DNA damaging agents, and AG14361 with two (Table 2). Vanillin, Nu7441, mirin and W7 all showed a weak synergistic effect with only one DNA damaging agent. These results were promising, given the *C*. *neoformans ku80Δ* mutant also exhibits slight sensitivity to phleomycin [13].

**Table 2:** FIC values were determined for each of the drugs selected. The ΣFIC was calculated as FIC A + FIC B where the combinations are considered synergistic when the ΣFIC value is <0.5, weakly synergistic when ΣFIC is 0.6 to 1.0, additive when ΣFIC is 1.0 to 2.0 and antagonistic when the ΣFIC is >2.

Inhibitor	DNA damaging agent ΣFIC		
	Mercaptopurine	Hydroxyurea	Phleomycin
**W7**	1.19	0.55	1.06
**Chlorpromazine**	0.73	0.88	0.94
**Vanillin**	0.79	1.19	1.71
**Nu7026**	0.55	0.94	0.73
**Nu7441**	0.86	1.10	1.09
**Mirin**	0.73	1.05	1.01
**SCR7**	0.80	0.81	0.88
**AG14361**	0.71	1.04	0.98

### Treatment with W7, chlorpromazine, NU7026, mirin and AG14361 enhance frequency of deletion of *ADE2*

Given that all eight tested inhibitors exhibited synergy with DNA damaging agents, all were trialed in biolistic transformation by including the inhibitor into the YPD + sorbitol plates used at the beginning of the protocol; the strain to be transformed is incubated on this media for four hours prior to transformation and recovers for four hours after. The fungus is therefore only exposed to the inhibitor for eight hours. Adding inhibitor in varying concentrations at this point would ensure disruption of NHEJ at the time of transformed DNA entry and integration. After recovery, the *C*. *neoformans* was transferred to G418 selective media lacking inhibitor, enabling reestablishment of NHEJ. Four concentrations per inhibitor were trialed, all less than the MIC (Table 1). In some instances, much lower concentrations were needed; for example, after no colonies would grow on high concentrations of NU7026, concentrations were subsequently dropped to 5, 2.5 and 1.125 μg/mL.

The first gene ever deleted *via* biolistic transformation in *C*. *neoformans* was the phosphoribosylaminoimidazole carboxylase-encoding *ADE2* gene, loss of function mutants of which are easily identified due to their pink colonies and adenine auxotrophy. We employed the same locus in our initial studies as we had data indicating it was unusually easily deleted [14], with ~35% of deletion construct transformants of the *ade2Δ* genotype.

**Fig 2:**
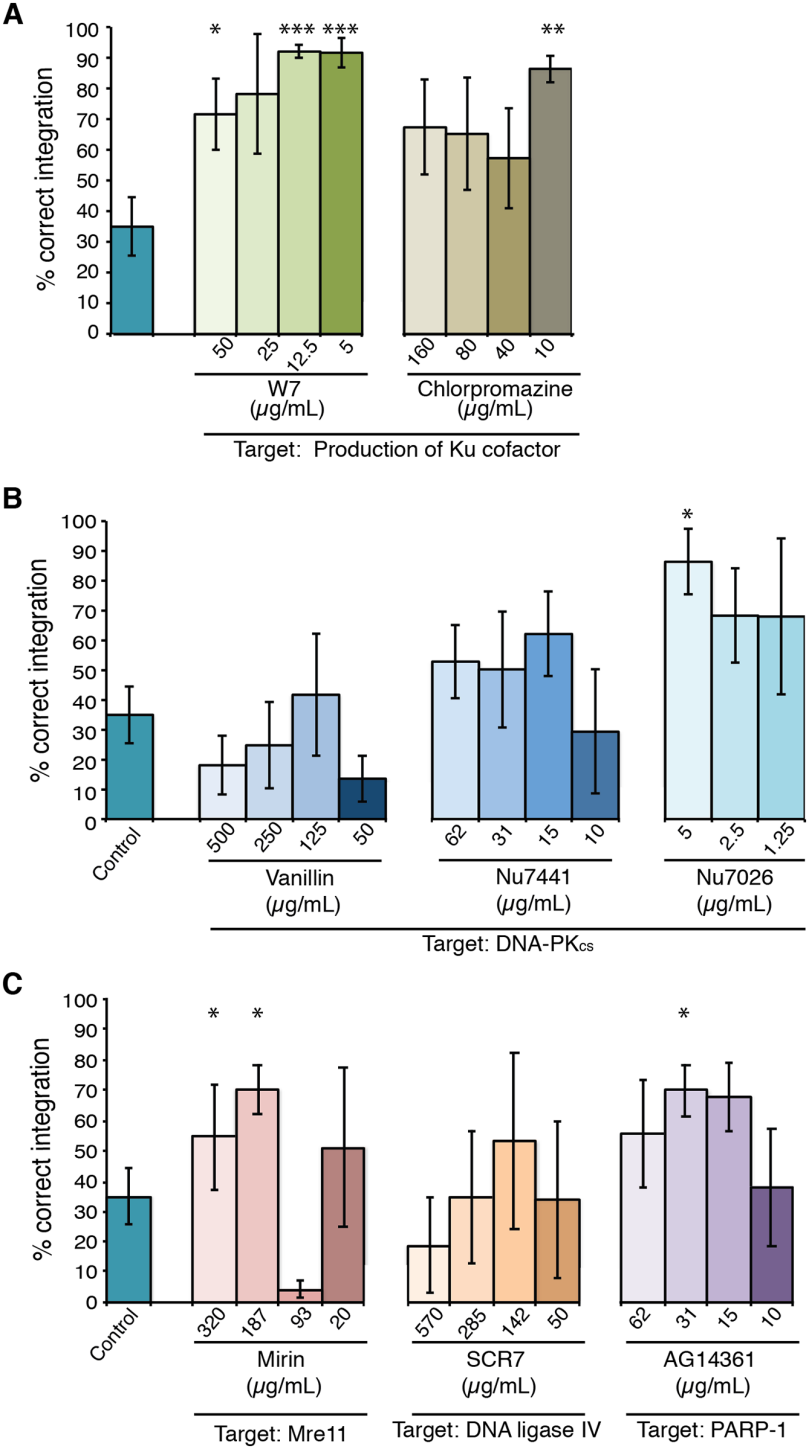
Inhibitors of mammalian NHEJ repair result in increased homologous integration in *C*. *neoformans* at the *ADE2* locus. **A.** Percentage correct integration of the *ADE2* construct comparing no inhibitor to the presence of two inhibitors of Ku cofactor production—W7 and chlorpromazine. **B.** Percentage correct integration of the *ADE2* construct in the presence of three inhibitors targeting DNA-PK_cs_−vanillin, Nu7441 and Nu7026. **C.** Percentage correct integration of the *ADE2* construct comparing no inhibitor to the presence of inhibitors of Mrell (mirin), DNA ligase IV (SCR7) and PARP-1 (AG14361). All values show mean, error bars show S.E.M. * = P<0.05, ** = P<0.01, *** = P<0.005.

Inclusion of either W7 or chlorpromazine, inhibitors of mammalian calmodulin proposed to inhibit NHEJ through depletion of Ku cofactor InsP_6_, resulted in a significant increase in the deletion of *ADE2* (Fig 2A). Treatment with W7 at either 5 or 12.5 μg/mL was particularly effective, resulting in deletion frequencies equivalent to those reported for a *ku80Δ* mutant [13]. A statistically supported increase in *ADE2* deletion was also observed, but to a lesser extent, for one of the three inhibitors of mammalian DNA-PKcs (Nu7026), the Mre11 inhibitor mirin and the PARP-1 inhibitor AG14361 (Fig 2B and 2C). Importantly, none of the strains obtained exhibited changes in growth rate or morphology. Furthermore, while we observed an increase in the proportion of correct integrants, there was no gross change in the total number of colonies obtained from each transformation event.

### Four of the inhibitors also showed an increase when using *LAC1* as a target

Our success in enhancing deletion of the *ADE2* gene was tempered by one significant fact; it is known that the rate of homologous recombination differs from locus to locus, and that *ADE2* is particularly easy to delete. To address concerns of locus specificity, we focused on the five most promising inhibitors—W7, chlorpromazine, NU7026, mirin and AG14361 –in biolistic transformations to delete the laccase-encoding *LAC1* gene [50] Each of the inhibitors were this time tested only at the optimal concentration observed in the previous experiment.

**Fig 3:**
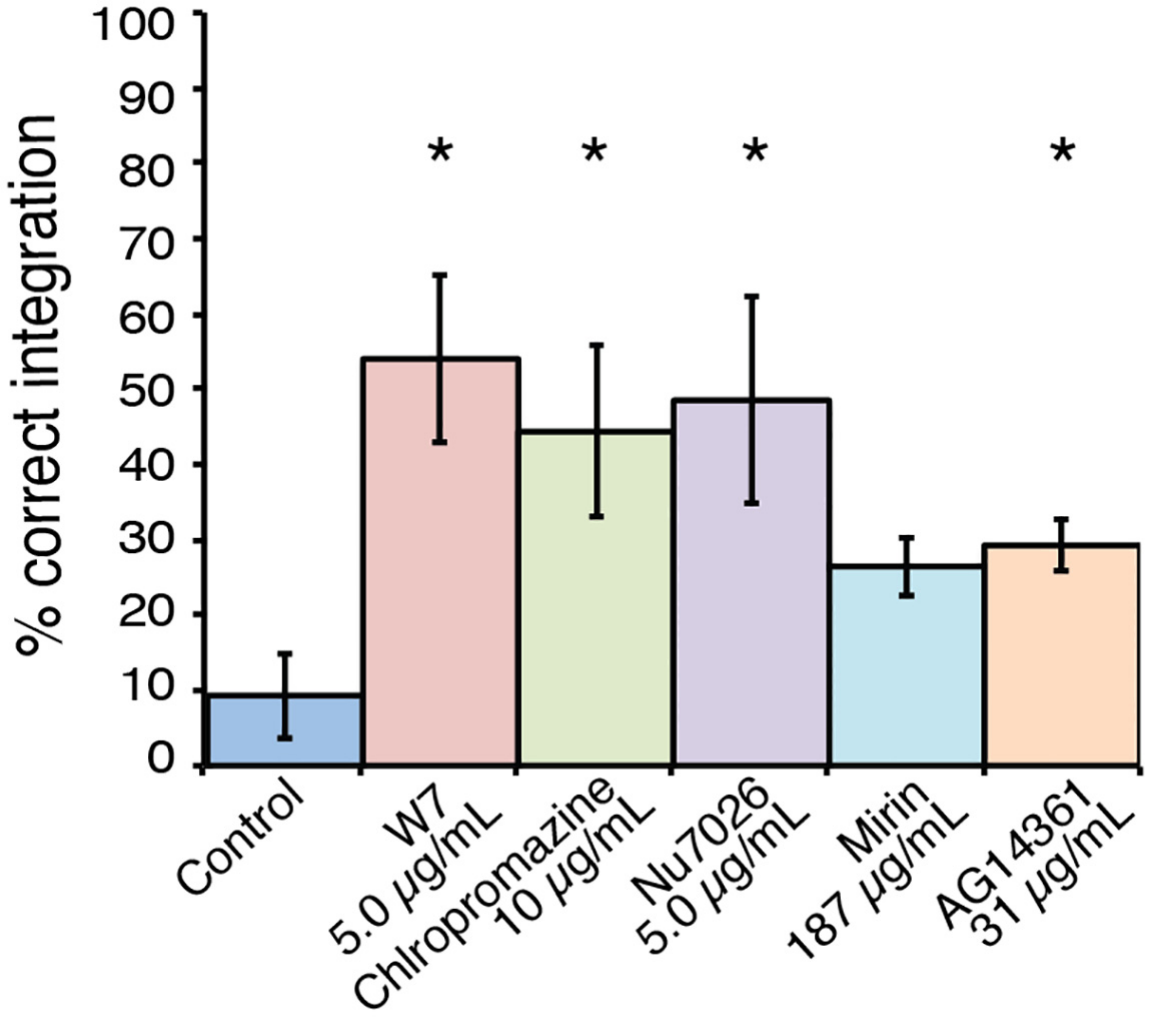
Inhibitors of mammalian NHEJ repair result in increased homologous integration in *C*. *neoformans* at the *LAC1* locus. Drug treatment resulted in a significant increase in the frequency of homologous integration events for four of the five inhibitors tested. All values show mean, error bars show S.E.M. * = P<0.05.

Without addition of any inhibitor, our control frequency for *LAC1* deletion was only ~10%; much lower than for *ADE2* and more in line with typical deletion frequencies observed in the field [11, 12]. Once again W7 and chlorpromazine performed as the best inhibitors, along with Nu7026. In contrast to the *ADE2* experiment, inhibitor treatment during *LAC1* deletion resulted in approximately half of all transformants exhibiting the deletion genotype, a 5-fold increase in frequency when compared to the no inhibitor control (Fig 3). The failure of mirin to yield a statistically significant result in the *LAC1* experiment could be due to the typically large fluctuations in the number of transformants observed during biolistic transformation.

## Discussion

Despite the clinical importance of *C*. *neoformans*, there have been few improvements over the last decade in the molecular tools available to characterize this species. The current methodologies have been invaluable in elucidating the function of many genes, however these techniques have their limitations and new advances are required to facilitate everyday experiments, particularly for large-scale genomic studies [51]. A good example of this is the recent publication of the sequence of *C*. *neoformans* var. *grubii* type strain H99, which represents one of the most complete eukaryotic genomes to date [52], due in no small part to the extensive transcriptomics performed to enable its annotation. Of the 6967 predicted protein coding genes, 45.5% (3170) are listed as hypothetical proteins. The functions of the 1197 miscRNAs also identified during transcriptome sequencing are likewise unknown. To enable a deeper understanding of these genes, as well as those with an annotation based purely on bioinformatic homology, a more robust procedure for performing molecular genetic modifications is essential.

Efforts to enhance the biolistic transformation procedure through disruption of NHEJ have had some success. The use of a *ku80Δ* strain can increase rates of recombination to nearly 100% [13], but recent publications have revealed that the Ku proteins may be playing a role during infection of the mammalian host. Liu and colleagues demonstrated that a *ku80Δ* mutant has diminished virulence in murine infection competition experiments [27], and Chen *et al*. discovered transcription of *KU80* is higher in cells isolated from the cerebrospinal fluid of an infected human than from cells grown on YPD [26]. Given these observations, it would be unwise to employ strains carrying the *ku80Δ* mutation during *in vivo* studies since it may influence the outcome of infection. Consequently, the *ku80Δ* mutant has seen little use by the community since its development a decade ago.

However the concept underlying the use of *kuΔ* mutants still has merit. By employing discoveries made in the field of mammalian NHEJ, we have been able to emulate the higher homologous recombination rates of the *ku80Δ* mutant without the experimental consequences of permanently compromising NHEJ at the genetic level. Of the eight inhibitors of mammalian NHEJ that we tested, four influenced the rate of homologous recombination for multiple loci. Notably, some of the best results observed correlated with employing the inhibitor at a concentration much lower than the MIC, and much lower than the concentration that confers increased sensitivity to DNA damaging agents. For example, W7 enhanced the rate of generation of a gene deletion at only 5 μg/mL, substantially lower than the concentration at which it displayed weak synergy with the DNA damaging activity of hydroxyurea.

Importantly, the use of transient drug treatment to enhance homologous integration efficiency is also highly cost effective. The best performing inhibitor, W7, is inexpensive and used at such low concentrations that it only costs around $USD 0.26 per plate; a negligible expense that significantly increases the chance of gaining a correct gene deletion on the first attempt, saving both time and reagents on repeated efforts to perform a simple genetic modification. We now employ W7 in all transformations in our laboratory, routinely achieving correct integration rates exceeding 50%.

In this work we demonstrated a new, easily applied methodology that will expedite acquisition of precise genetic alterations in *C*. *neoformans* that has distinct advantages over current protocols. We have successfully employed multiple inhibitors to reproducibly enhance the deletion rate at multiple loci. Based on this success, we anticipate that the use of these inhibitors will not only become widespread in the *Cryptococcus* community, but may also find use in other fungal species as well. More importantly, they will greatly facilitate the ongoing efforts designed to elucidate the function of all genes in the *C*. *neoformans* genome, enabling a much deeper understanding of the process of pathogenesis in this clinically important species.

## Supporting Information

S1 Table Primers used in this study. (DOCX)
