# Supplementary material for: Chemical Inhibitors of Non-Homologous End Joining Increase Targeted Construct Integration in Cryptococcus neoformans
Source: PLoS One. 2016 Sep 19;11(9):e0163049. doi: 10.1371/journal.pone.0163049 (PMC5028063; doi:10.1371/journal.pone.0163049)
Supplement: S1 Table — (DOCX) [file pone.0163049.s001.docx]

**Supplementary Table 1: Primers used in this study**

| Primer ID | NAME | Sequence |
| --- | --- | --- |
| UQ234 | *ADE2* deletion construct | GCTGCGAGGATGTGAGCTGGAGAGCG |
| UQ235 | *ADE2* deletion construct | GGTTTATCTGTATTAACACGGAAGAGATGTAG |
| UQ1439 | *ADE2* deletion construct | GAGTTAAAGTGTCGATGGCAG |
| UQ1440 | *ADE2* deletion construct | CCAGCTCACATCCTCGCAGCTTTGCTACAAGGGGTGCGGATG |
| UQ1441 | *ADE2* deletion construct | CCGTGTTAATACAGATAAACCATTGGTGCGATATCTGTAACT |
| UQ1442 | *ADE2* deletion construct | CGCTTAGGACAAGAGAGGCTA |
| UQ3686 | *LAC1* deletion construct | GCAGGTATGACCACAATCTTC |
| UQ3687 | *LAC1* deletion construct | TCCAGCTCACATCCTCGCAGCTACTGTGAGTGTCGGTATAGC |
| UQ3688 | *LAC1* deletion construct | GCTATACCGACACTCACAGTAGCTGCGAGGATGTGAGCTGGA |
| UQ3689 | *LAC1* deletion construct | CCAATCCAAGCTATAGTCACGGTTTATCTGTATTAACACGGA |
| UQ3690 | *LAC1* deletion construct | TCCGTGTTAATACAGATAAACCGTGACTATAGCTTGGATTGG |
| UQ3691 | *LAC1* deletion construct | GGAGGATGACGAGGACTCTGA |
